# Paradoxical euthyroid hormone profile in a case of Graves' disease with cardiac failure

**DOI:** 10.1186/1687-9856-2011-8

**Published:** 2011-07-24

**Authors:** Ganesh Jevalikar, Priyanka Gupta, Vijayalakshmi Bhatia, Aditya Kapoor, Sanjay Gambhir

**Affiliations:** 1 Department of Endocrinology, Sanjay Gandhi Postgraduate Institute of Medical Sciences, Raebareli Road, Lucknow (U.P.), 226 014, India; 2 Department of Cardiology, Sanjay Gandhi Postgraduate Institute of Medical Sciences, Raebareli Road, Lucknow (U.P.), 226 014, India; 3 Department of Nuclear Medicine, Sanjay Gandhi Postgraduate Institute of Medical Sciences, Raebareli Road, Lucknow (U.P.), 226 014 India

## Abstract

Cardiac failure is an uncommon complication of juvenile hyperthyroidism. We describe an adolescent boy with Graves' disease who developed manifestations of heart failure while on antithyroid medications. There was no evidence of any underlying cardiac disease. He had paradoxical euthyroid hormone profile which rose to hyperthyroid range when the manifestations of the cardiac failure subsided. The case highlights several unusual features of Graves' disease.

## Introduction

Thyroid hormones have multiple effects on the cardiovascular system [1]. Although many of the characteristic signs and symptoms of hyperthyroidism are cardiovascular, cardiac failure is seen only in up to 6% cases [2] and is more common in older age with underlying ischemic or hypertensive cardiomyopathy [1,2]. There are very few reports in the literature of cardiac failure in juvenile hyperthyroidism in the absence of underlying heart disease [3].

A state of cardiac failure is known to cause changes in the thyroid hormone profile, especially low total tri-iodothyronine (T3) [4,5]. This phenomenon of non-thyroidal illness syndrome (NTIS) may be attributable to various mechanisms including changes in hypothalamic-pituitary axis, altered thyroid hormone binding and altered de-iodinase activity [6]. The same phenomenon has not been well documented in cardiac failure due to hyperthyroidism.

Herein we describe an adolescent boy who presented in cardiac failure due to Graves' disease and had a paradoxical euthyroid profile.

## Case Report

A 13 year old boy presented with palpitations of six months duration, fever and hyperdefecation for a month and generalized edema since three days. Fatigue, diaphoresis, tremors, polyphagia and weight loss were present for six months. He was diagnosed to have hyperthyroidism five months before presentation to us. He was started on carbimazole 15 mg daily at the time of diagnosis, which was increased to 45 mg daily one week prior to presentation at our hospital.

On examination, the patient was febrile with a heart rate of 130 per minute and a blood pressure of 140/60 mm Hg. There was generalized edema and raised JVP (12 cm of water). He had exophthalamos. The thyroid gland was diffusely enlarged to approximately 60 grams and a bruit was heard over the thyroid. Cardiomegaly was present as well as a grade 3/6 apical ejection systolic murmur. There was mild weakness (grade 4 power) of hips, knees and shoulders, with hyperreflexia. Hepatosplenomegaly was present. Hemoglobin was 96 g/L (normal, 130-160 g/L), total leukocytes 4.1 × 10^9^/L (normal, 4.5-13.5 × 10^9^/L), and platelets 51 × 10^9^/L (normal, 150-400 × 10^9^/L). He had hyponatremia (serum Na 121 mEq/L; normal,135-145 mEq/L) and hypoalbuminemia (serum albumin 25 g/L; normal, 35-55 g/L). Blood and urine cultures, Widal test and smear for malarial parasite were negative. The ECG showed sinus tachycardia, normal QRS voltages and T wave inversion in precordial leads V2-V6. The chest radiograph was normal except for mild cardiomegaly (cardiothoracic ratio 54%). The echocardiogram showed mild pulmonary arterial hypertension, dilated right ventricle and tricuspid regurgitation with normal contractility of both the ventricles. There was no evidence of underlying congenital or acquired heart disease.

Thyroid function tests revealed low T3 (0.77 nmol/L; normal, 1.3-2.8 nmol/L), normal total T4 (104.1 nmol/L; normal, 60-160 nmol/L) and free T4 (22.6 pmol/L; normal, 10-25 pmol/L) with a suppressed TSH (<0.15 mU/L; normal, 0.3-5 mIU/L). Thyrotropin receptor antibody titer was 28.5 IU/L by ELISA (normal, <1.5 IU/L).

In addition to the supportive care, the patient was started on prednisolone 60 mg/day, propranolol 40 mg/day and carbimazole was continued. After three days of treatment, the signs of heart failure subsided; however, fever and tachycardia were persistent. Repeat T4 and free T4 now rose to hyperthyroid levels (Table 1), with serum albumin of 31 g/L. He was given potassium iodide drops for further symptomatic improvement. During the hospital stay he developed hyperglycemia probably caused by the combined effect of hyperthyroidism and glucocorticoid therapy, requiring insulin for two weeks.

**Table 1:** Serial thyroid functions, clinical features and treatment

Day	T4 (60-160 nmol/L)	Free T4 (10-25 pmol/L)	T3 (1.3-2.8 nmol/L)	TSH (0.3-5 mIU/L)	Albumin (35-55 g/L)	Clinical event	Treatment
1	104.8	22.6	0.7	<0.15	25	CHF*	Carbimazole Prednisolone Propranolol
4	205.3	58.0	1.7		31	Resolution of CHF*	
8	135.0	49.3				Persistent fever and tachycardia	KI^† ^started
12			1.0			Fever resolved	Steroid tapering started
4 week	80.8				36		Off KI^† ^and steroids
9 week	35.0					Clinically hypothyroid	Carbimazole dose reduced RaIA^¶ ^done
4 month	119.8			<0.15	49	Clinically euthyroid	
9 month**	<12.9			>60		Radiation induce Hypothyroidism	Thyroxine started

*CHF: congestive heart failure, ^† ^KI: Potassium iodide, ^¶ ^RaIA: Radioactive Iodine Ablation

**: The patient was off carbimazole for 2 months at the time of this follow up.

After three days of starting potassium iodide, the fever subsided and there was significant improvement in signs of thyrotoxicosis. The steroids and potassium iodide were tapered and omitted sequentially. Thyroid hormone levels gradually normalized after four weeks of treatment. A repeat echocardiogram showed mild mitral and tricuspid regurgitation, normal left ventricular contractility and right ventricular systolic pressure of 33 mm Hg (normal, <30 mm of Hg) suggestive of mild pulmonary hypertension.

Because of severe presentation and poor access to medical assistance from his native place, he was subjected to radio-iodine ablation after two months of presentation to us, with 10 mCi of radioactive I^131^. The thyroid scan done at this time revealed diffuse increase in tracer uptake. In subsequent follow up he was diagnosed to have radio-iodine induced hypothyroidism requiring thyroxine replacement (Table 1).

## Discussion

Hyperthyroidism has multiple effects on the cardiovascular system including decreased systemic vascular resistance and increased resting heart rate, left ventricular contractility and blood volume leading to a state of high cardiac output [1]. A small percentage of patients with hyperthyroidism may present with clinical manifestations of cardiac failure [2].

Our patient was admitted with signs and symptoms suggestive of hyperthyroidism and cardiac failure. There was no evidence of underlying heart disease on clinical examination and echocardiography. The chest X-ray and ECG findings did not suggest primary pulmonary pathology or viral myocarditis. In a study of 591 adults with hyperthyroidism, heart failure was reported in 5.8% cases [2]. Atrial fibrillation increases the risk of cardiac failure but it can also occur in sinus rhythm [7]. There are very few reports of cardiac failure in juvenile hyperthyroidism. In a retrospective analysis of 21 children with hyperthyroidism, cardiac failure was observed in 2 patients [3]. There is some evidence of presence of thyrotoxic cardiomyopathy in children [8].

In our patient, in the presence of normal left ventricular contractility, edema, raised JVP and hepatomegaly could be attributed to the pulmonary hypertension and right ventricular failure. In contrast to the fall in systemic vascular resistance, pulmonary arterial hypertension has been reported in as many as 47% of patients with hyperthyroidism [9]. The various underlying mechanisms are related to increased venous return due to increased blood volume, high output or autoimmune pulmonary endothelial injury or increased metabolism of certain vasodilator substances [10]. The increased blood volume (postulated to occur as a result of activation of renin-angiotensin-aldosterone system and increased red cell mass) causes volume overload of the right ventricle, which when unaccompanied by fall in pulmonary vascular resistance, leads to right ventricular failure and systemic venous congestion [10,11].

The thyroid hormone profile of the patient revealed low T3 and paradoxically normal total and free T4. The hormones rose to hyperthyroid levels when the cardiac failure subsided. This can be explained by mechanisms similar to those causing thyroid function abnormalities in non-thyroidal illnesses, also known as sick euthyroid syndrome. It is a well known phenomenon in various diseases including cardiac failure [4,5]. The various underlying mechanisms include impaired hypothalamic TRH secretion, decreased binding proteins, decreased de-iodinase activity, presence of inhibitors of thyroid hormone binding, impaired tissue uptake and altered receptor expression [6]. The most common manifestation of this is a low T3. However, low T4 can also be seen in severe or prolonged illness [12]. Another reason for lower than expected T3 and T4 in our case could be the low albumin levels. Per se albumin has little influence on hormone levels, unless associated with changes in thyroglobulin and transthyretin. As all three are synthesized in liver, albumin level may serve as a surrogate marker for thyroglobulin [13]. Free T4 results in non thyroidal illness are variable and are dependent on the assay method used [6]. In our case the free T4 was measured by a solid phase radioimmunoassay and equilibrium dialysis method was not used. Free T3 measurements have been shown to have a modest decline in levels, however reliable estimation of free T3 is difficult [6].

The phenomenon similar to sick euthyroid syndrome in hyperthyroidism has been previously documented in relation to myocardial infarction, diabetic ketoacidosis, pneumonia and fulminant hepatitis [14]. However to the best of our knowledge this is the first report of this phenomenon in cardiac failure due to juvenile hyperthyroidism.

In addition to non thyroidal illness, other factors like prior use of carbimazole and concurrent iodine deficiency can influence the results of the thyroid function. Patients with hyperthyroidism on carbimazole therapy tend to normalize T4 ahead of T3, the latter being a better indicator of the clinical status. Hence, some patients may have normal T4 despite being clinically hyperthyroid [15]. However, T3 is usually high in such cases (isolated T3 toxicosis). The presence of low T3 and the rise of T4 and free T4 to hyperthyroid range on the same dose of carbimazole ruled out this possibility in our patient.

The finding of normal T4 may also be seen in hyperthyroidism with co-existing iodine deficiency [16]. These patients have higher frequency of isolated T3 toxicosis. Since our patient consumed iodized salt, this mechanism was not invoked to explain the lower than expected T4.

The severity of the case required the use of glucocorticoids and potassium iodide for control of the symptoms. Glucocorticoids reduce de-iodination of T4 to T3. In addition, T4 secretion may be reduced due to direct thyroidal action and reduced thyroid stimulating antibodies [17]. Potassium iodide rapidly reduces the release of T4 and T3 from the thyroid gland and suppresses iodide oxidation and organification. When used for short term, it is useful for rapid symptomatic relief. To avoid stimulation of thyroid hormone synthesis, iodide should be given not earlier than 60 minutes after the start of the anti-thyroid medication [18].

Some other less common features of Graves' disease noted in this case were leukopenia, thrombocytopenia [19], splenomegaly [20] and persistent fever [21].

## Key Messages

• Congestive cardiac failure occurring in the absence of atrial fibrillation or underlying heart disease is a rare manifestation of hyperthyroidism.

• Occurrence of cardiac failure in hyperthyroidism may be associated with a paradoxically euthyroid hormone profile due to the phenomenon of sick euthyroid syndrome.

## Consent

Written informed consent was obtained from the patient for publication of this case report. A copy of the written consent is available for review by the Editor-in-Chief of this journal.

## Competing interests

The authors declare that they have no competing interests.

## Authors' contributions

GJ prepared the manuscript. VB critically reviewed and modified the manuscript. All authors were closely involved in care of the patient and interpretation of the unusual presentation and course. All authors read and approved the final manuscript.
